# Supplementary material for: Are carbon emissions trading and green financial instruments synergistic? -Comprehensive quantitative research based on content analysis
Source: PLoS One. 2024 Mar 7;19(3):e0298601. doi: 10.1371/journal.pone.0298601 (PMC10919601; doi:10.1371/journal.pone.0298601)
Supplement: S1 Data — (ZIP) [file pone.0298601.s001.zip › Data/地级市Z_F工作报告.zip!/░▓╗╒/┘±╓▌╩╨/└┤╫╘╣1⁄2╓┌║┼í╛┬φ┐╦╩2╛▌═°í┐_2010.docx]

各位代表：
　　现在，我代表市人民政府，向大会报告工作，请予审议，并请市政协委员和其他列席人员提出意见。
　　一、抢抓机遇，迎难而进的2009年
　　刚刚过去的2009年，是我市改革发展进程中极不寻常、很不平凡的一年。一年来，我们坚持以邓小平理论和“三个代表”重要思想为指导，深入学习实践科学发展观，在中共亳州市委的坚强领导下，在市人大、市政协的监督支持下，坚持“发展为上、民生为先、稳定为重，少说多做、真抓实干”，与全市人民一道，抢抓中央扩大内需和省委、省政府加快皖北发展的双重机遇，应对金融危机和严重自然灾害的双重挑战，扎实做好“保增长、保民生、保稳定”的各项工作，经济社会继续保持较好发展势头，圆满完成市二届人大五次会议确定的各项目标任务。
　　初步预计，全市实现生产总值450亿元，比上年增长12。5%，连续4年保持两位数增幅；财政收入30。9亿元，增长28。3%；财政支出80亿元，增长31。3%；固定资产投资175亿元，增长51。4%；社会消费品零售总额190。8亿元，增长18。8%；进出口总额1。6亿美元，增长43。9%；城镇居民人均可支配收入14040元，农民人均纯收入4000元，分别增长10%和11。6%，主要经济指标增速位居全省前列。谯城区、涡阳县、蒙城县生产总值超过百亿元，增速达到12%以上。
　　一年来，我们主要抓了以下工作：
　　（一）以保增长为首要任务，狠抓结构调整，经济发展取得新成效。
　　工业经济快速增长。积极推进工业强市战略，加快实施“125行动计划”和“工业经济三年倍增计划”，全市净增规模工业企业119家，亿元企业达到60家；规模以上工业实现增加值85亿元，增长27%以上；工业对经济增长的贡献率提高到43。8%。三次产业比例由2008年的29。5：30。3：40。2调整为27。7：32。9：39。4，二产比重提高2。6个百分点。抓住国家重大产业政策调整机遇，相继制定一系列规划，促进主导产业的调整与振兴。《安徽省（亳州）现代中药产业规划》顺利通过省级评审；《亳州市煤炭开采及综合利用规划》启动实施，刘店煤矿正式投产，全市已探明煤炭储量增加到43。6亿吨、居全省第3位；制定了《亳州市农产品加工业发展规划》和《亳州市白酒产业调整与振兴规划》，工业发展后劲进一步增强。加快科技进步，亳州现代中药特色产业基地被纳入科技部“火炬计划”，成为全省自主创新政策支持的重点；亳州中药材进出口检测中心通过国家认证；济人药业“疏风解毒胶囊”被列入国家医保目录，被卫生部指定为防甲流首选中药。加强节能减排工作，完成了省政府下达的约束性指标。
　　农村经济不断壮大。坚持以农民增收为核心，全面落实各项支农惠农政策，开展小麦高产攻关活动，实施玉米振兴计划，农业在50年不遇的大旱之年喜获丰收，粮食总产89亿斤、增产5亿斤、占全省新增10亿斤的一半，小麦单产位居全省第1位，涡阳县荣获“全国粮食生产先进县标兵”称号。实施畜牧业振兴计划，肉蛋产量分别增长10%和9。3%，规模养殖比重突破40%。开展农业产业化“532”提升行动，农民专业合作组织发展到580家，市级以上龙头企业增加到178家。强化农民技能培训和转移就业服务，农民工资性收入增长9%。
　　服务业发展步伐加快。蒙城汽贸中心入驻汽车4S店8家，销售收入突破30亿元。亳州建材大市场建设顺利，亳州宾馆已经开工。《亳州旅游发展总体规划》和《中华药都？养生亳州行动计划》正式启动，三大体系建设和30项重点工作扎实推进，保健食品、保健酒投放市场，养生专业街建设开始实施，8集专题片《走遍中国？走进亳州》制作成功并在央视播放，文化旅游公司挂牌成立，古井酒文化博览园成功创建国家4A级旅游景区，实现国内旅游总收入18。2亿元，同比增长62。9%。对外贸易逆势上扬，进出口海关A类管理企业达到8家，出口增速居全省第1位。
　　（二）以统筹发展为主要目标，强化基础设施建设，城乡面貌呈现新变化。
　　城市承载能力进一步提升。强力推进25平方公里的市南部新区建设，修建主干路网21公里，配套设施正在完善；10多家学校、医院、机关、事业单位正在规划建设，新的城市发展框架初具雏形；工业组团排定入区项目22个，总投资16。2亿元，已开工建设项目10个，3个项目即将竣工投产。备受关注的10项重点工程中，涡河景观带一期工程、北关历史街区配套完善工程、曹操纪念馆建设等6项工程已经竣工，博物馆建设等4项工程完成年度任务。开工建设涡北水厂，改造供水支管网，新增供水干支管网101公里，铺设燃气管网40公里。中心城区累计投入财政性建设资金12。53亿元。
　　城镇发展空间布局进一步优化。启动市区、涡阳县、利辛县总体规划修编，完成蒙城县总体规划、南部新区控制性详规和41个乡镇规划编制工作，开展新一轮土地利用总体规划修编，城市规划、建设和管理工作得到加强。积极争取上级支持，多渠道增加投入，办成了一些多年想办而没有条件办的大事。亳州火车站改造工程主体框架完工，泗许高速公路（亳州段）建设加快，阜周高速公路北段建成通车，商杭客运专线列入铁道部和安徽省铁路建设规划，许昌—亳州—宿州铁路、济宁—祁门高速公路前期工作进展顺利，京九铁路动车组即将开通，交通条件明显改善。
　　农村面貌进一步改观。经过全市人民的共同努力，涡河近期治理工程顺利通过竣工验收；新改建县乡公路217公里，完成村村通公路845公里，建设农村客运站场4个；完成120个村配电网改造，新建农村沼气池2。4万口，改水、改厨、改厕7。5万户，新解决28万农村人口饮水安全问题；完成人工造林6。5万亩，新建农田林网85万亩；积极推进新农村建设示范工程，涌现出曹市镇高长营村、西潘楼镇于寨村、牛集镇宋老家村、许疃镇许疃村等一批新典型。
　　（三）以“三个活动年”为抓手，完善体制机制，改革开放带来新活力。
　　坚持招商是“第一要事”，扎实开展“招商引资推进年”活动。成功举办了2009年药博会，积极参加中博会、高交会等大型推介活动，集中签约项目55个，签约金额142亿元。四大煤业集团入驻亳州，亳州众和煤业公司挂牌运营，注册资金50亿元的安徽亳州煤业公司正式成立，东方希望饲料开始试产，和记黄埔、江中制药等大型企业已经落户，重庆啤酒、捷众制药、蜀中制药等项目开工建设，与广药集团、天津医药、三精制药等一批知名企业合作积极推进。全市累计利用市外内资187。2亿元、同比增长99。4%，实际利用外资1。16亿美元、增长36。1%。
　　坚持项目是“第一抓手”，扎实开展“项目建设推进年”活动。全年共谋划储备项目857个，总投资1780亿元；争取中央新增投资项目315个，总投资19。1亿元，落实中央和省补助资金6。9亿元。实施2000万元以上的市重点管理项目186个，完成投资102亿元，同比增长134。6%。主动与央企对接合作，签订合同、意向项目11个，总投资263。41亿元。完成5个省级开发区共扩区10平方公里任务，固定资产投资增速居全省第3位。
　　坚持环境是“第一要素”，扎实开展“发展环境优化年”活动。推行为企业服务全程代理制，建立领导干部联系企业制度，对21家企业实行驻点帮扶，成立金融为企业服务中心，为137家企业办理了土地证、房产证，帮助中小企业融资10。1亿元。向县区派驻挂职金融副县区长，开展金融机构负责人帮扶企业工作，组织银企对接会5次，落实资金62。4亿元。实行重点工作“一票认可”、“一票否决”制，开展“民主评议百名科长”和模拟仿真督查机关效能活动，始终保持治理发展环境的高压态势。
　　坚持改革是“第一动力”，深入推进改革创新。政府机构改革顺利实施，市政府工作部门减少到33个，“事改企”工作全面推进，扩权强镇试点取得积极成效。国有企业改革进一步深化，古井集团40%国有股权成功转让上海浦创投资公司，双轮集团被深圳盈信投资公司收购，使企业迸发出新的活力。投融资体制进一步完善，企业上市前期工作积极推进，市建投公司首次成功发行企业债券10亿元；全市实现土地出让金收益25。25亿元，其中市本级7。35亿元。金融改革进程加快，谯城区农村信用社通过改制即将挂牌为亳州药都农村合作银行，徽商银行入驻亳州，工商银行亳州支行升格为二级分行，组建小额贷款公司5家；全市政策性农业保险稳步推进，种植业承保面积1132。28万亩，养殖业承保24。43万头；全年新增贷款65。15亿元，是上年的2。8倍，是前7年的总和。完成集体林权制度改革，开展农村土地流转，全市复垦整理土地8613亩，新增建设用地6250亩。全民创业步伐加快，新增私营企业1。17万家，个体工商户2。25万户，非公有制经济占GDP的比重提高到51%。
　　（四）以改善民生为根本，全面加强社会建设，人民生活水平得到新提高。
　　民生工作扎实推进。全面完成26项民生工程，共投入资金19。7亿元，其中落实配套资金2。8亿元。强化创业扶持和就业帮扶，城镇新增就业岗位3。1万个，新转移农村劳动力5。7万人，城镇登记失业率控制在4%以内。职工基本养老、基本医疗、失业、工伤、生育保险超额完成任务。新建、改扩建农村敬老院50所，五保集中供养率达到25。2%。发放城乡居民最低生活保障金2。2亿元，救助城乡低保人员25万人，享受城乡医疗救助17万人次，城市人均住房面积低于14平方米的低保家庭实现应保尽保。
　　各项社会事业全面进步。教育事业健康发展，亳州师专新校区、亳州一中南校区、亳州九中开工建设，亳州职业技术学院在校生超过6000人，全市高中阶段毛入学率突破60%、比上年提高4。9个百分点。公共卫生服务体系不断完善，城镇居民基本医疗保险和新型农村合作医疗覆盖率达到90%，基层医药卫生体制综合改革试点工作正式启动，市人民医院新院顺利开工、南院投入使用，重大传染病防治取得明显成效，利辛县甲型H1N1流感防控工作有力、有序、有效，实现无重症、无死亡、无扩散目标。文化事业不断进步，花戏楼改扩建工程完工，华佗中药博物馆、曹操纪念馆建成开放。广播影视、新闻出版、文学艺术事业进一步发展。成功举办了亳州首届中国健身气功博览会暨华佗五禽戏养生健身节，市体育场开工建设。低生育水平持续稳定，人口自然增长率控制在8。34‰。民族工作获省政府民族团结进步表彰。
　　坚持稳定是第一责任，推进“平安亳州”建设，加强社会治安综合治理，认真开展“打黑除恶”、打击“两抢一盗”专项行动，严厉打击经济领域违法犯罪活动，处置非法集资专项斗争取得决定性胜利，社会治安形势保持稳定。完善矛盾纠纷排查调处机制，开展信访积案化解年活动，信访秩序明显好转。全面建立应急管理体系，安全生产形势总体平稳，产品质量和食品药品市场专项整治深入推进。积极支持驻亳部队建设，国防动员、民兵预备役、人民防空和双拥优抚工作取得新成绩。人事、编制、外事、侨务、统计、物价、气象、地质、防震减灾、无线电管理工作扎实推进，档案、地方志、对台工作和妇女儿童、老龄、慈善、残疾人事业取得新进展。
　　在推进经济社会发展过程中，我们高度重视政府自身建设，坚持依法行政，推进管理创新，狠抓工作落实。深入开展学习实践科学发展观活动，突出实践特色，解决实际问题。自觉接受人大法律监督、工作监督和政协民主监督，密切与工会、共青团、妇联等人民团体的联系，虚心听取各方面意见和建议。认真办理人大议案、代表建议和政协委员提案，办结率100%，满意率96。8%。落实政府信息公开条例，推进政务公开、村务公开和电子政务，政府工作效率和透明度不断提高。坚持从严治政，规范权力运行，加强反腐倡廉建设，强化行政监察和审计监督，惩治和预防腐败体系不断完善，政风建设取得新成效。
　　各位代表，在看到成绩的同时，我们也清醒地认识到全市经济社会发展还面临不少困难和问题。主要是：农业基础较为薄弱，农民增收乏力；工业发展滞后，投资总量偏小，人才资源匮乏，发展后劲不足；社会管理和公共服务历史欠账较多，城市形象和功能需要进一步提升；征地拆迁难度加大，维护稳定任务还很艰巨；政府自身建设与跨越发展的要求还有差距，执行力、落实力尚待进一步提高。对此，我们一定高度重视，积极采取措施，认真加以解决。
　　总的来说，2009年，是我市加快发展、顽强崛起，各项工作取得重大进展的一年；是亳州全面提速、奋力赶超，在全省的地位和影响显著提高的一年；是全市人民创造力、凝聚力、战斗力明显增强，干部群众心气最顺的一年。一年来，我们深切体会到：必须善抓机遇、顺势而为，牢牢把握工作主动权；必须善于统筹、科学运作，坚持市场调节与政府调控有机结合，推动经济社会协调发展；必须勇于创新、敢闯敢试，把上级政策与本地实际有机结合，创造性地开展工作。实践充分证明：事业是干出来的，成绩是拼出来的；亳州有大发展的条件，亳州人有干成事的能力；只要我们始终保持干事创业的责任心，始终保持昂扬向上的精气神，咬定目标，敢于超越，永不停步，亳州的未来一定会更加美好。
　　各位代表，过去一年取得的成绩，是省委、省政府和市委正确领导的结果，是市人大、市政协监督支持的结果，是全市人民共同奋斗的结果。在此，我代表市人民政府，向全市工人、农民、知识分子、广大干部，向各民主党派、工商联、无党派人士、人民团体，向驻亳人民解放军指战员、武警部队官兵、公安政法干警，向所有关心支持我市改革开放和现代化建设的各界人士，表示衷心的感谢并致以崇高的敬意！
　　二、阔步前行，充满希望的2010年
　　2010年是地级亳州市组建10周年，也是完成“十一五”规划，实现“一个赶超、三个翻番、城市面貌大变样”目标的最后一年。当前，我们面临的仍然是一个机遇与挑战并存、机遇大于挑战的环境。从全国看，经济发展环境总体好于去年，但宏观形势仍然十分复杂，经济发展中的不确定、不可预料因素增多；中央继续实施积极的财政政策和适度宽松的货币政策，同时稳中有调；国家将加大对中西部地区、民生领域、社会事业、“三农”、节能环保、居民消费等方面的支持力度，对我们总体有利。从全省看，去年全省生产总值超过万亿元，投资总额超过9000亿元，财政收入超过1500亿元，支持皖北地区发展的能力增强；“皖江城市带承接产业转移示范区”规划即将实施，为我们对接长三角提供了广阔空间。从我市看，加速发展的态势日趋强劲，认识更加统一，思路更加清晰，基础正在夯实，各方面有利条件明显增多，为实现奋力崛起提供更加有力的支撑。只要我们树立必胜信心，不为任何风险所惧，不为任何干扰所惑，抢抓机遇，乘势快进，就一定能够在新的起点上把改革发展稳定的各项工作做得更好。
　　今年市政府工作的总体要求是：以邓小平理论和“三个代表”重要思想为指导，深入学习实践科学发展观，全面贯彻落实党的十七大、十七届四中全会和中央经济工作会议精神，坚持“发展为上、民生为先、稳定为重，少说多做、真抓实干”，继续开展“项目建设推进年、招商引资推进年、发展环境优化年”活动，扩大经济总量，调整优化结构，转变发展方式，确保快速增长，力求争先进位，实现经济社会又好又快发展。
　　今年经济社会发展的主要预期目标是：生产总值增长12%以上；财政收入增长20%；固定资产投资增长40%以上；社会消费品零售总额增长16%以上；进出口总额增长20%以上；城镇居民人均可支配收入增长10%；农民人均纯收入增长8%；城镇登记失业率控制在4%以内；人口自然增长率控制在8。5‰以内；居民消费价格涨幅4%左右；COD排放量控制在11100吨以内，二氧化硫排放量控制在6800吨以内；万元生产总值能耗降低4。5%。
　　完成上述目标任务，我们将注重把握好以下几点：一是瞄准赶超的目标不动摇。坚持发展是第一要务、招商是第一要事、落实是第一真功，强基固本、能快则快、加速发展。二是落实行之有效的好思路。抓住土地、融资、园区、招商等几个关键环节，优化生产要素配置，提高统筹运作能力，重点实施、整体推进。三是珍惜来之不易的好势头。把握宏观环境新变化，强化工业发展主动力，加快重大产业和基础设施建设，统筹城乡发展，确保民生改善、社会稳定。四是保持争先进位的好状态。强化等不得的紧迫感、慢不得的危机感和坐不住的责任感，不甘落后，真抓实干，争取各项主要经济指标在全省的位次不断前移。
　　2010年，着重抓好以下六个方面工作：
　　（一）坚持工业为本，促进工业发展大跨越。
　　工业兴则亳州兴。继续实施“工业经济三年倍增计划”、“125行动计划”，着力培育壮大现代中药、能源化工、汽车制造、食品加工等支柱产业，引进和发展新兴产业，力争年内完成工业投资100亿元以上，实现规模工业增加值100亿元以上，新增规模工业企业100家以上，力争年销售收入超亿元企业突破70家。
　　壮大支柱产业。着眼打造千亿元中药产业，大力实施《安徽省（亳州）现代中药产业规划》和“1125”药企振兴计划，全面提升产业发展水平，引进一批知名药企落户亳州，争取规模药业企业达到100家，力争3年内药业产值超过150亿元。着眼打造千万吨煤炭基地，大力实施《亳州市煤炭开采及综合利用规划》，确保袁店、板集煤矿建成投产，加快建设“586”煤化工项目，努力实施板集电厂建设工程，推进耿皇、信湖、花沟、赵集煤矿及涡北循环经济园前期工作。着眼打造百亿元白酒产业，大力实施《亳州市白酒产业调整与振兴规划》，力争白酒业产值增长30%以上。着眼打造500亿元农产品加工业，大力实施《亳州市农产品加工业发展规划》，建设食品工业园，力争农产品加工业产值增长30%以上。积极推进安驰公司与江淮汽车集团的合作，实现优势互补，做大做强，带动零部件生产，壮大汽车产业。
　　推进自主创新。主动承接泛长三角地区产业转移，建成一批科技企业孵化器，争取全年新增省级企业技术中心3家、高新技术企业8家、高新技术产品6个。以科技进步为支撑，加强产学研合作，鼓励药企购买药方、验方50个以上，积极筹建中国中医科学院中药研究所亳州分所，采取股份制等形式成立中国中药材检测中心，争取获得科技部现代中药产业集群项目支持。积极发展低碳经济，做好节能减排工作，全面完成“十一五”期间我市GDP综合能耗下降20%的目标任务。
　　完善服务体系。健全中小企业信用担保体系，推进中小企业融资服务平台建设；落实税收优惠政策，扩大失业保险基金支出范围；加强创业辅导中心建设，提升完善创业基地10个；加强企业人才引进工作，举办高级管理人员培训班，在全市选择20家企业开展管理规范化试点；放手发展非公经济，帮助他们解决实际困难和问题，争取全年新增个体工商户2。2万户、中小企业2000家。继续开展全民创业活动，坚持能人创企业、百姓创家业、干部创事业，形成支持创新、宽容失败、鼓励冒尖的社会氛围。
　　（二）统筹城乡发展，推动“三农”工作大提升。
　　提高农业综合生产能力。着眼打造百亿斤粮仓，继续实施小麦高产攻关活动和玉米振兴计划，建立优质小麦原料基地160万亩。抓好大型商品粮基地、亚行贷款农业综合开发项目、现代农业综合开发示范区项目建设，支持涡阳县创建“小麦亩产千斤县”。推进农业标准化建设，促进中药材种植向三县辐射，力争面积扩大到75万亩、其中标准化基地面积5万亩以上，争取2-3个品种通过GAP认证。发展设施蔬菜，推进蔬菜标准园区建设。实施畜牧业振兴计划，强化畜禽防疫，规模养殖比重提高5个百分点以上。加强农业科技服务，提高农业机械装备水平。
　　推进农业产业化发展。开展土地流转经营权抵押贷款试点，力争年底全市土地流转总面积达到60万亩，加快形成规模化种养、区域化布局、集约化经营的发展新格局。鼓励农产品加工龙头企业参与土地流转，扩大生产规模，提高市场份额，新增省级龙头企业3-5家。加快农民专业合作社建设，开展横向联合，实现优势互补，争取全年新增农民专业合作社120家。
　　加快农村城镇化步伐。强力推进农村土地整治工作，开展城乡建设用地置换，促进农民向中心村镇集中、农业向适度规模经营集中、工业向园区集中。加强村镇规划管理，推进文明集镇建设，逐步在每个乡镇新建或完善一条街、一个农贸市场、一个居住小区，在扩权强镇试点镇新建或完善一个工业小区、一个污水处理厂、一个垃圾处理厂（焚烧炉）。完成全市村庄建设规划，引导农民按规划改造旧房、建设新居，切实解决农民建房难、乱建房问题。
　　改善农村生产生活条件。加强农田水利基本建设，实施中小河流治理和25座大中型病险涵闸除险加固工程，争取洼地治理项目；积极开展植树造林，完善林业产业体系和生态体系，确保森林覆盖率达到18%以上；改造县乡公路200公里，全面完成“十一五”规划的“村村通”建设任务，实现建制村通水泥（油）路目标；继续实施改水、改厕、沼气和安全饮水项目。推动医疗卫生、教育培训、新闻出版、广播电视、现代通讯、体育健身等公共服务向农村延伸。
　　（三）突出发展旅游和现代物流，推进服务业结构大调整。
　　突出发展文化旅游业。大力实施《中华药都·养生亳州行动计划》，加快建设养生专业街，规划建设中华药都养生园；实施以花戏楼为核心的北关历史街区（古城文化旅游区）改造项目，完成花戏楼国家4A级旅游景区创建工作；推进涡阳县老子文化生态园、蒙城县庄子文化园、利辛县西淝河生态水乡旅游项目；建设高星级饭店，开发城区温泉资源；办好2010年药博会、老子文化节和首届中国亳州白酒养生文化节，开展“长寿之星”评选活动；强化宣传推介，拓展客源市场，着力提升亳州旅游的整体吸引力，确保全年旅游总收入增长50%以上。
　　加快发展商贸物流业。加强专业批发市场和物流中心建设，提升中药材交易中心、蒙城汽贸中心、亳州农产品交易中心辐射能力，筹建农资大市场、农机专业市场、现代中药物流园区项目；引进家乐福、沃尔玛、大润发等大型商贸企业集团，做大做强本地龙头企业。继续推进“家电下乡工程”、“万村千乡市场工程”和“新网工程”，实施农村社区综合服务体系建设项目，扩大农村消费。加强物流基础设施和城市商业网点建设，发展电子商务、连锁经营、特许经营、物流配送等现代流通方式。鼓励发展餐饮住宿、购物娱乐、信息咨询、服务外包等行业，满足社会多样化消费需求。着力打造中药材、农副产品、汽车出口基地，培育进出口超2000万美元企业1家、超1000万美元企业6家，力争中药产业进出口突破1亿美元。
　　大力发展房地产业。出台鼓励房地产业发展的政策措施，逐步放开户籍限制，吸引农民进城买房。引进知名房地产企业，开发建设中高档住宅小区，合理引导住房消费。加大经济适用房、廉租房建设力度，进一步改善低收入家庭的居住条件。按照节能、降耗、环保的要求，搞好住房设计和建设。完善房地产交易、中介、物业管理服务体系，促进房地产业健康发展。
　　积极发展金融业。大力实施《亳州市金融业三年发展规划》，积极引进外资、合资及其它股份制金融机构，加快建立多元化金融服务体系。引导银行业金融机构牢固树立服务意识，多形式创新金融产品，最大限度地支持地方经济发展。启动蒙城县农村信用社改制工作，组建1—2家村镇银行、5—8家小额贷款公司、2—3家信用担保公司。争取设立亳州现代中药发展基金，推进企业上市工作取得实质性进展。
　　（四）改善人居环境，加快城市大建设。
　　加快城市基础设施建设。年内实施亳州城区建设项目56个，投资22。6亿元。其中，投资52680万元，精心打造南部新区：实施古井大道、淮河路、汤王大道、连接线跨铁路立交桥引道和泵站工程；继续建设工业组团道路，尽快形成南部新区路网框架；实施道路绿化工程，提升南部新区形象。投资1364万元，完善提高建成区：继续实施希夷大道、芍花路、魏武大道人行道改造工程；完善红绿灯等交通安全设施，对城市主次干道路灯进行维修改造。投资16965万元，加快“两水”工程建设：启动涡北污水处理厂建设工程，完成涡南污水处理厂深度处理技术改造工程；继续实施涡北自来水厂工程，完善第三水厂供水设施，提升城市供水能力。加强老城区的保护与利用：改造北关历史街区主要街巷建筑立面，综合整治历史建筑。稳妥实施“城中村”改造，积极推进城区坑塘治理，抓好城区菜市场、停车场、小绿地、小游园、公厕、垃圾中转站等配套设施建设，做好小街小巷美化亮化工作。
　　重点实施市区10大建设工程：1。继续推进南部新区扩区及路、水、电、气等基础设施建设；2。建设亳州一中南校区和亳州九中；3。完成魏武大道硬化、亮化、美化综合改造工程；4。开工建设建安大桥和105国道涡河大桥；5。建设儿童福利院、流浪未成年人救助保护中心；6。加快南部新区安置区、汤王大道还原小区、人民西路还原房和廉租房、经济适用房建设，完成建筑面积30万平方米；7。改造完善城区供水和污水处理系统，提高供排水能力；8。开工建设市图书馆、档案馆；9。开工建设华佗广场；10。开工建设华佗中医院新院。
　　加快县城开发建设。实施“123”城建工程，即每个县城改造和建设10公里城区样板路，新增开发区面积2平方公里以上，新增城市公共绿地30万平方米。实施一批城建重点工程：涡阳县重点建设体育场、垃圾处理场、中医院迁址等5项工程；蒙城县重点建设第一人民医院住院大楼、市民文化园、庄周集中安置区等5项工程；利辛县重点实施城区水环境治理、人民医院新区、皖北商城等5项工程。
　　提升城市管理水平。加强土地收储工作，清理规划区内闲置土地，加大拆违力度，严格控制新生违法建筑。创新城管综合执法方式，下放管理权限，强化基层责任。加强市容环境卫生管理，切实解决占道经营、乱停乱靠等问题。大力弘扬亳州城市精神，积极开展文明创建活动，争创省级园林城市和全省文明城市。
　　（五）突出招商引资，以大投入促大发展。
　　坚持不懈开展“招商引资推进年”活动。牢固树立算大账、看长远的发展共赢意识，以大开放的胆识和胸怀吸引大企业、大项目；落实招商引资项目推进工作机制，严格执行一会准入、一次性告知、一站式审批、一个窗口收费、一次性验收、一次兑现制度，对重大招商项目实行“一事一议、一企一策、特事特办”；坚持产业招商，成立药业、农业产业化、房地产业、文化旅游产业四大产业招商中心；推行专业招商，在长三角、珠三角、京津地区设立招商分局，人员从市直单位选派或从派驻地亳州籍人士中选聘，吸引客商来亳投资兴业；严格目标考核，继续坚持招商引资调度述职制度，落实招商引资与单位办公经费挂钩制度，确保全年招商引资额增长40%以上。
　　坚持不懈开展“项目建设推进年”活动。继续坚持督查调度、目标考核和重大项目领导分包责任制。抓好项目谋划，最大限度地争取上级资金扶持，争取列入省“861”行动计划项目260个以上，总投资1100亿元以上，年度投资100亿元以上、增长1倍以上。完成火车站改造工程，加快推进亳州至宿州铁路、亳州至蚌埠铁路、济宁至祁门高速公路、省道307线亳州段一级公路改建和引淮济涡等重大基础设施项目前期工作，争取商杭铁路客运专线项目及早开工，积极配合禹州至亳州铁路建设。进一步加快中烟公司蒙城雪茄烟生产部扩建工程进度。按照产业集群、园区集聚、发展集约的思路，集中优势资源，加快市南部新区工业组团建设，新增入驻企业50家；培育建设特色产业园区和乡镇工业功能区，争取入园企业100家以上。加大城乡电网建设投资，优化线路走向，构建坚强、安全电网，为经济发展提供电力保障。
　　（六）加强社会建设，实现民生大改善。
　　以实施33项民生工程为抓手，让全市人民得到更多实惠。落实更加积极的就业政策，新增2。64万个就业岗位，实现下岗失业人员再就业7200人，新转移农村富余劳动力5。6万人。健全社会保障体系，推动城镇居民基本医疗保险和工伤保险市级统筹，稳步开展蒙城县新型农村基本养老保险试点工作。逐步完善社会救助体系，加强城乡低保工作，保障困难群体生活。进一步提高五保供养水平，集中供养率达到35%。
　　大力发展各项社会事业。加快义务教育发展，推进标准化学校建设和校舍安全工程，做好亳州师专搬迁和亳州职业技术学院迎接教育部复评工作，筹建华佗技师学院，建设职教园区，加强教师队伍建设，提高教育教学质量。积极推进医药卫生体制改革，确保谯城区综合改革试点成功，加快市人民医院新院区和乡镇卫生院标准化建设，加强重大传染病防控。引进高级人才，设立博士后工作站。实施文化体制改革，筹建五禽戏馆、梆剧团剧场，做好五禽戏、古井酿造技艺等非物质文化遗产申报和文物保护工作，争取古井酿造遗址列入国家级文物保护单位。开展全民健身运动，参加省第12届运动会。关爱农村留守儿童，筹建五位一体的科技文化艺术中心（妇女儿童活动中心、青少年活动中心、职工之家、科技馆、文化馆）。加强出生人口性别比综合治理，做好流动人口计生管理与服务工作，提高出生人口素质，稳定低生育水平。积极开展双拥共建活动，做好国防动员、民兵预备役、拥军优抚和人防工作。组织实施第六次全国人口普查，做好新闻出版、民族、宗教、外事、侨务、对台、地方志、气象、价格、防震减灾、无线电管理工作，重视和推动妇女儿童、老龄、慈善和残疾人等事业发展。
　　维护社会和谐稳定。严格落实信访责任制，畅通信访渠道，着力排查化解矛盾纠纷，加强普法宣传和人民调解工作。强化社会治安综合治理，深入开展平安创建工作，依法打击各类违法犯罪活动，坚决打击非法集资、传销、诈骗行为，进一步提高执法的公信力，努力创造和谐稳定的社会环境。加强应急管理，提高处置突发公共事件能力。扎实做好安全生产工作，坚决遏制重特大事故的发生。整顿和规范市场秩序，加强食品药品监管，深入开展药品药材市场综合整治，打造诚信药都。
　　各位代表，在加强上述六个方面工作的同时，市政府经过认真调查研究，确定了今年必须做好的10项重点工作：1。确保完成工业、投资、财政三年倍增计划，主要经济指标增速保持全省先进位次；2。继续开展“招商引资推进年、项目建设推进年、发展环境优化年”活动，招商引资、固定资产投资增长40%以上；3。完成“中华药都·养生亳州”行动计划和“1125”药企振兴计划年度目标，力争产值超过80亿元；4。推进煤电、煤化工项目建设，尽快形成1000万吨煤炭生产能力；5。实施《亳州市白酒产业调整与振兴规划》，确保古井集团白酒年销售收入突破20亿元，加快打造100亿元白酒生产基地和以古井镇为中心的酒文化圣地；6。实施大型商品粮基地和现代农业综合开发示范区项目；7。实施中小河流治理和25座大中型病险涵闸除险加固工程，争取西淝河洼地治理项目；8。加快建设市南部新区，确保工业组团入驻企业新增50家；9。加强投融资平台建设，实现融资10亿元以上，新增贷款80亿元以上；10。全面实施33项民生工程，确保完成年度目标任务。
　　三、加强政府自身建设，不断提高行政能力
　　实现全年的目标任务，最根本的是坚定不移地贯彻落实科学发展观；最关键的是加强政府自身建设，提高经济调控和社会管理能力；最重要的是继续倡导求真务实的工作作风，以良好的精神状态推动工作落实。
　　（一）加强学习，提高科学发展能力。全体政府工作人员特别是各级领导干部要强化学习理念，把学习作为一种责任和使命，加快知识储备和更新，不断适应新形势、新任务的要求。全面把握科学发展观的丰富内涵和精神实质，深刻领会中央、省经济工作会议精神，找准结合点，进一步增强工作的前瞻性、预见性和主动性，不断提高领导发展、推动发展、服务发展的能力。
　　（二）依法行政，提高民主决策能力。进一步增强人大意识和政协意识，执行市人大及其常委会的各项决议、决定，提高人大代表建议和政协委员提案的办理质量，加强与工青妇等群众团体的紧密联系，严格按法定权限和程序行使权力、履行职责。健全科学民主决策机制，建立问计于民的长效机制，规范政府信息公开，完善政府新闻发布制度，加强市长热线和行风热线建设，推进市政府网在线访谈活动制度化，使政府决策更贴近民情、体现民愿、顺应民心。
　　（三）转变作风，提高执行落实能力。坚持把抓落实作为各级政府工作人员的基本功，围绕重点工作和重大项目，建立专题工作推进制度，强化抓落实的责任，健全抓落实的机制，做到时时在状态、天天有激情，真正形成主动落实有氛围、跟踪落实有督查、考核落实有问责的良好局面。继续推行为民为企服务全程代理制，深化“民主评议百名科长”和模拟仿真督查机关效能活动，强化责任追究，严肃查处不作为、慢作为、乱作为，坚决杜绝有令不行、有禁不止的现象，不断增强政府的公信力、执行力和推动力。
　　（四）求真务实，提高廉洁从政能力。切实改进文风和会风，最大限度地减少各类礼仪性、应酬性活动，把主要精力用在抓落实、抓发展上，用在为基层、为群众服务上。弘扬艰苦奋斗、勤俭节约的作风，坚决反对铺张浪费，把更多的财政资金投向民生领域、公益事业和困难群体。注重惩防并举，进一步落实廉政建设“一岗双责”制度，扎实推进以完善惩防腐败体系为重点的反腐倡廉建设。加强行政监察、财政监督、审计监督，严肃查处违法违纪案件。
　　各位代表，机遇稍纵即逝，形势催人奋进。让我们在中共亳州市委的坚强领导下，进一步解放思想，抢抓机遇，团结拼博，克难奋进，努力保持经济社会又好又快发展的良好势头，以优异成绩迎接地级亳州市组建10周年！
　　名词解释：
　　“1125”药企振兴计划——力争全市药业生产企业达到100家以上，通过三年努力，到2012年，年销售收入5亿元以上的企业达到10家，2亿元以上的20家，1亿元以上的50家，药业生产企业产值达到150亿元以上。
